# HIF1-Alpha Expression Predicts Survival of Patients with Squamous Cell Carcinoma of the Oral Cavity

**DOI:** 10.1371/journal.pone.0045228

**Published:** 2012-09-18

**Authors:** Marcelo dos Santos, Ana Maria da Cunha Mercante, Iúri Drumond Louro, Antônio José Gonçalves, Marcos Brasilino de Carvalho, Eloiza Helena Tajara da Silva, Adriana Madeira Álvares da Silva

**Affiliations:** 1 Programa de Pós Graduação, Faculdade de Ciências Médicas da Santa Casa de São Paulo, São Paulo, São Paulo, Brazil; 2 Laboratório de Biologia Molecular, Hospital Heliópolis, São Paulo, São Paulo, Brazil; 3 Departamento de Anatomia Patológica, Hospital Heliópolis, São Paulo, São Paulo, Brazil; 4 Núcleo de Genética Humana e Molecular, Departamento de Ciências Biológicas, Universidade Federal do Espírito Santo, Vitória, Espírito Santo, Brazil; 5 Departamento de Biologia Molecular, Faculdade de Medicina de São José do Rio Preto, São José do Rio Preto, São Paulo, Brazil; 6 Departamento de Biologia, Universidade Federal do Espírito Santo, Alegre, Espírito Santos, Brazil; Okayama University, Japan

## Abstract

**Background:**

Oral squamous cell carcinoma is an important cause of death and morbidity wordwide and effective prognostic markers are still to be discovered. HIF1α protein is associated with hypoxia response and neovascularization, essential conditions for solid tumors survival. The relationship between HIF1α expression, tumor progression and treatment response in head and neck cancer is still poorly understood.

**Patients and Methods:**

In this study, we investigated HIF1α expression by immunohistochemistry in tissue microarrays and its relationship with clinical findings, histopathological results and survival of 66 patients with squamous cell carcinoma of the lower mouth.

**Results:**

Our results demonstrated that high HIF1α expression is associated with local disease-free survival, independently from the choice of treatment. Furthermore, high expression of HIF1α in patients treated with postoperative radiotherapy was associated with survival, therefore being a novel prognostic marker in squamous cell carcinoma of the mouth. Additionally, our results showed that MVD was associated with HIF1α expression and local disease relapse.

**Conclusion:**

These findings suggest that HIF1α expression can be used as a prognostic marker and predictor of postoperative radiotherapy response, helping the oncologist choose the best treatment for each patient.

## Introduction

Head and neck cancer is a significant cause of mortality and morbidity worldwide, presenting approximately 600,000 new cases yearly [1], whereas tumors of the oral cavity show 389,000 new cases per year, with a mortality rate of 50% [2].

Currently, the most important prognostic factor is the presence of regional lymph node metastases, which correlates with a 50% reduction in life expectancy [2]–[4]; however, micrometastases may not be detected by routine histology [5].

Oral squamous cell carcinoma is a solid tumor that relies on a hypoxia cellular response system for tumor progression [6]–[12]. Focal hypoxia is found in the majority of solid tumors due to quantitative and qualitative alterations in tumor vasculature, leading to local reduction of oxygen availability [13].

Tumoral response to radiotherapy has been studied through hypoxia measurements in cervical cancer, as well as other tumors, including head and neck tumors [14], [15]. Hypoxia-inducible factor-1 (HIF1) is a heterodimeric transcriptional complex that functions as the main regulator of systemic and cellular oxygen homeostasis [16]–[25]. When activated, HIF1 can induce the transcription of over 60 genes, as an attempt to avoid hypoxia-mediated dell death. Among HIF1-regulated genes, there are angiogenic and proliferating factors, glucose transporters, anaerobic glycolytic enzymes and others, that are important for tumorigenesis [25]–[28].

Expression of HIF1α has been studied in renal, bladder, colorectal, breast, ovary and cervical tumors and it was often associated with patient prognosis [29]–[36]. Positive HIF1α expression has been associated with improved prognosis in head and neck tumor patients that underwent surgery [37]. Additionally, Fillies and cols, described a better prognosis for patients with high HIF1α expression in squamous cell carcinomas of the tongue basis treated with radiotherapy [38]. Nonetheless, lower survival and higher disease relapse in irradiated patients has been associated with strong HIF1α protein expression, as reported by Aebersold et al. [39]. These contradicting results indicate a high complexity of the hypoxia signaling pathway and its participation in radiotherapy treatment response.

A tempting hypothesis to explain these observations would envision HIF1α activation as an inducer of higher tumor vascularization and oxygenation due to Vascular Endothelial Growth Factor (VEGF) expression [40], which would ultimately increase the concentration of intratumoral reactive oxygen species after radiotherapy and therefore render such tumors more responsive to this type of therapy.

In this study, we demonstrate that high HIF1α expression is associated with local disease-free survival. Moreover, in patients treated with postoperative radiotherapy, high HIF1α expression was associated with survival, therefore being a novel prognostic marker in squamous cell carcinoma of the oral cavity. We also show that microvessel density (MVD) is associated with HIF1α expression and local disease relapse. These findings suggest that HIF1α expression can be used as a prognostic marker and a tool for choosing the best treatment for each patient.

## Materials and Methods

### Ethics

The present study was approved by the Ethics Committee of the Heliopolis Hospital on 06/10/2008 (CEP # 619).

### Sample

Samples were collected by the Head and Neck Genome Project (GENCAPO), a collaborative consortium created in 2002 with more than 50 researchers from 9 institutions in São Paulo State, Brazil, whose aim is to develop clinical, genetic and epidemiological analysis of head and neck squamous cell carcinomas. In this study, we analyzed 66 parafinized tumor samples of squamous cell carcinomas of the lower mouth, surgically treated in the Head and Neck Surgery Department of the Heliópolis Hospital, São Paulo, Brazil, during the period of January/2001 to December/2007. Exclusion criteria were: previous surgical or chemotherapy treatment, presence of distant metastasis, no removal of cervical lymph nodes and positive surgical margins.

Postoperative radiotherapy was indicated when the tumor invaded adjacent tissues (pT4) or cervical lymph nodes were compromised (pN+). Histopathologycal characteristics of all samples were revised by A.M.C.M. (pathologist,author) of the Heliópolis Hospital. According to TNM classification (3^rd^ edition) [41], 27 tumors were T1 and T2, 13 tumors were T3 and 26 tumors were T4. Thirty six cases showed metastasis to cervical lymph nodes. Well differentiated tumors were found in 30 samples, moderately differentiated tumors in 31 and poorly differentiated in 5 (Table 1).

**Table 1:** Correlation of tumor epidemiological and pathological features with HIF1α expression.

Features			HIF1α expression				
	Frequency		Weak		Strong		*p value*
	No.	(%)	No.	(%)	No.	(%)	
**Gender**							
Female	10	(15.2)	–	–	–	–	–
Male	56	(84.8)	–	–	–	–	–
**Age, yr (median 55, df ±10,9)**							
≤55	33	(50.0)	–	–	–	–	–
>55	33	(50.0)	–	–	–	–	–
**Treatment**							
Only operated	33	(50.0)	–	–	–	–	–
Operated and irradiated	33	(50.0)	–	–	–	–	–
**Site**							
Tongue	26	(39.4)	–	–	–	–	–
Inferior gums	12	(18.2)	–	–	–	–	–
Floor of the mouth	22	(33.3)	–	–	–	–	–
Retromolar area	6	(9.1)	–	–	–	–	–
**Stage**							
I+II	16	(24.3)	8	(23.5)	8	(25.0)	0.394
III	15	(22.7)	10	(29.4)	5	(15.6)	
IV	35	(53.0)	16	(47.1)	19	59.4)	
**Tumor size (pT)** *							
pT1, pT2	27	(40.9)	14	(41.2)	13	(40.6)	0.284
pT3	13	(19.7)	9	(26.4)	4	(12.5)	
pT4	26	(39.4)	11	(32.4)	15	(46.9)	
**Lymph node status (pN)** *							
Absent	30	(45.5)	16	(47.1)	14	(43.8)	0.787
Present	36	(54.5)	18	(52.9)	18	(56.2)	
**Differentiation grade**							
Well	30	(45.4)	20	(58.8)	10	(31.2)	0.079
Moderately	31	(47.0)	12	(35.3)	19	(59.4)	
Poorly	5	(7.6)	2	(5.9)	3	(9.4)	
**Lymphatic invasion**							
Absent	21	(31.8)	11	(32.4)	10	(31.3)	0.923
Present	45	(68.2)	23	(67.6)	22	(68.7)	
**Perineural invasion**							
Absent	31	(47.0)	17	(50.0)	14	(43.3)	0.611
Present	35	(53.0)	17	(50.0)	18	(56.7)	
**Total**	**66**	**(100.0)**	**34**	**(51.5)**	**32**	**(48.5)**	

* TNM classification (3^rd^ edition).

A gender and age characterization of the 66 patients showed a predominance of males (85%) and age varying from 34–81 years, with a mean age of 55 years. According to the anatomical localization of the tumor, 26 (39.4%) were on the tongue, 12 (18.2%) on inferior gums, 22 (33.3%) on the floor of the mouth and 6 (9.1%) on the retromolar area (Table 1).

Postoperative radiotherapy was indicated for 36 patients, but 3 deceased before the end of treatment and were excluded from the survival after radiotherapy analysis. After a follow up of at least 24 months, 36 patients (54.5%) were alive, 27 (41.0%) died due to the disease and 3 (4.5%) died of other causes. Local reoccurrence was observed in 23 cases (34.8%).

### Tissue Microarray

Formalin-fixed, paraffin-embedded tissue sections from 66 primary oral squamous cell carcinomas treated at the Head and Neck Surgery Department of Heliópolis Hospital, São Paulo, SP, were used for immunohistochemistry (IHC) analysis. Histological characterization of all samples was done by Hematoxylin and Eosin staining, followed by immunohistochemistry analysis of tissue microarrays (TMA). Two 1 mm cylinders taken from tumor central regions were used to represent each sample in the TMA slide (Beecher Instruments®, Silver Spring, MD, USA).

### Immunohistochemistry

Anti-HIF1α polyclonal antibody (Millipore Corporation®, USA) was used in the IHC reaction, at a 1∶150 dilution [42]–[44]. Positive (breast cancer controls) and negative (absence of primary antibody) controls were used. Sample scoring was performed by semi-quantitative microscopic analysis, considering the number of stained cells and signal intensity. Two spots were evaluated for each sample and a mean score was calculated. Considering the percentage of HIF1α immune-positive tumor cells, a score of 1 was given when ≤10% of cells were positive; 2 when 10–50% of cells were positive and 3 when ≥50% of cells were positive. Signal intensity was scored as negative (0), weak (1), moderate (2) and strong (3). Both scores were multiplied [45], [46] and the resulting score was used to categorize HIF1α expression as negative (≤1), weak (1–6) and strong (>6).

Angiogenic activity was assessed by MVD analysis using anti-CD34 antiboby (Santa Cruz Biotecnology®, USA) for the IHC reaction, at a 1∶150 dilution. Endothelial cell cytoplasmic staining was considered the positivity criterion. MVD was scored in four areas of the tissue array and categorized as ≤20, 20–40 and >40%. These analyses were performed by A.M.C.M. (pathologist, author).

### Statistical Analysis

The chi square and Fisher exact tests were used for association analysis and confirmation was obtained by the Lilliefors test (significance considered when p<0.05). Multivariate logistic regression was used to obtain odds ratio (OR) and confidence intervals (CI ≥95%). Survival was calculated by the number of months between surgery and death for each patient or the last appointment in case patients were alive. In order to calculate disease-free survival, the time endpoint was the date of local disease relapse. The Kaplan-Meier model was used for survival analysis, using the Wilcoxon p-value and the Cox Proportional Hazards to adjust p-values and obtain hazard ratio (HR). Statistical calculations were performed using the Epi Info® v3.4.3, 2007 and Statsoft Statistica® v7.0.61.0 softwares.

## Results

HIF1α expression was detected in all 66 tumors. However, expression was considered weak in 34 samples (51.5%), strong in 32 (48.5%) and it did not show association with tumor characteristics, such as size (*p* = 0.284), positive lymph nodes (*p* = 0.787) and others (Table 1).

In spite of being more frequent in surviving cases, strong expression of HIF1α did not show a significant correlation with the status alive (*p* = 0.094), but showed a significant association with cases with no local disease relapse (*p* = 0.002, Table 2). Multivariate analysis, considering pTNM, showed that HIF1α weak expression is an independent marker for local disease relapse, representing an increased risk of over 7 times in relation to strong expression (OR = 7.59, CI = 1.94–29.75).

**Table 2:** HIF1α expression association with the status alive or local disease relapse.

Prognostic features	HIF1α Expression				
	Weak		Strong		*p value*
	No.	(%)	No.	(%)	
**Survival status**					
Alive	15	(46.9)	21	(67.7)	0.094
Deceased	17	(53.1)	10	(32.3)	
**Local disease relapse**					
No	14	(43.7)	23	(82.1)	0.002
Yes	18	(56.3)	5	(17.9)	

**Figure 1:**
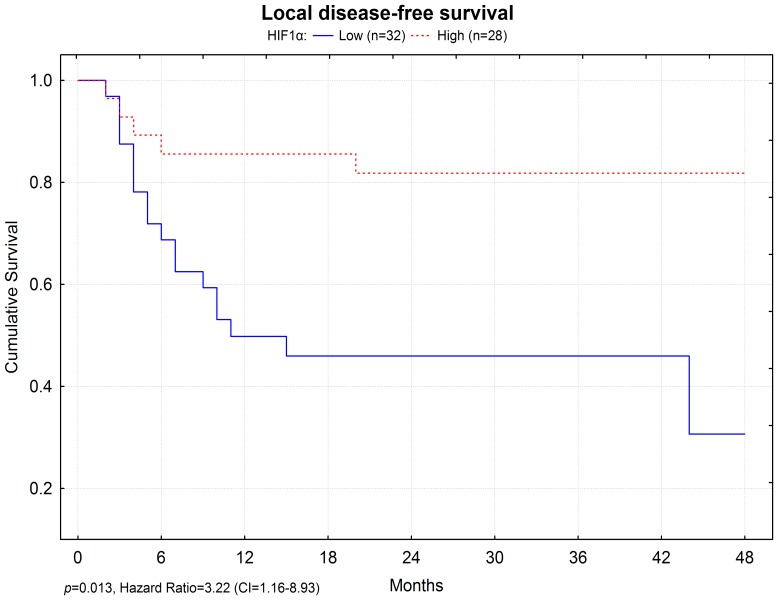
Local disease-free survival relation to HIF1α expression. High HIF1α expression is related to an increased local disease-free survival.

Although overall survival did not show a significant association with HIF1α expression intensity (*p* = 0.185), a strong expression was associated with local disease-free survival (*p* = 0.013, Figure 1). According to a 12 month after surgery follow up, approximately 10% of cases with high HIF1α expression showed local disease relapse, as compared to approximately 50% of relapse in patients with low HIF1α expression (Figure 1). Multivariate analysis, considering pTNM, revealed that a weak expression of HIF1α is an independent marker for a faster local disease relapse, with a 3-fold increased risk when compared to strong expression (HR = 3.22, CI = 1.16–8.93).

HIF1α low expression was associated with increased local disease relapse, independently from the choice of treatment (*p* = 0.038 for operated and irradiated patients; *p* = 0.039 for operated, but not irradiated patients), increasing the risk of relapse 11 times, both for operated and irradiated patients, as well as operated, but not irradiated cases (OR = 11.47 for operated and irradiated patients; OR = 11.48 for operated, but not irradiated patients. pTNM was considered in both analysis).

**Figure 2:**
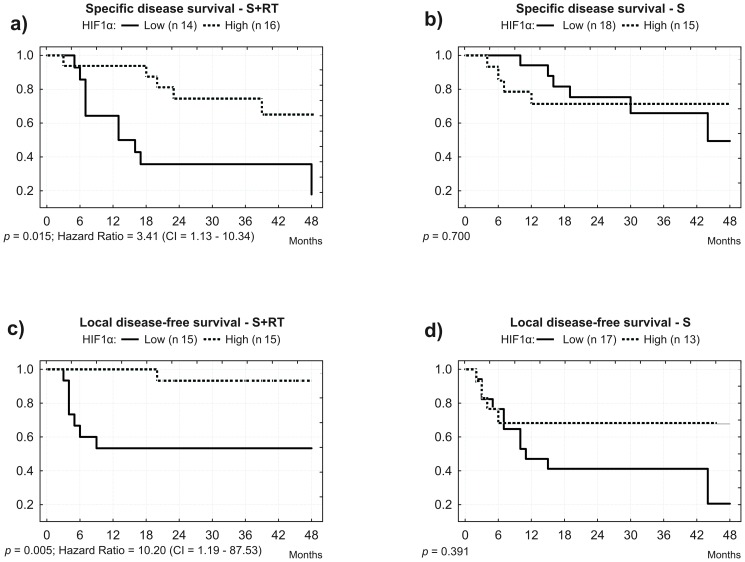
HIF1α expression and specific or local disease-free survival after treatment. Considering surgical (S) treatment only, high HIF1α expression predicts local disease-free survival. Considering surgery plus radiotherapy (S+RT), high HIF1α expression predicts both specific and local disease-free survival.

Most interestingly, when analyzing patients that undertook postoperative radiotherapy, low HIF1α expression correlated with a six-fold increased risk of death when compared to high expression (OR = 6.13, IC = 1.18–31.94, *p* = 0.031, considering pTNM). In contrast, surgically treated patients that did not make use of postoperative radiotherapy did not show this association (*p* = 0.366). Moreover, patients treated only with surgery showed no survival or local disease relapse difference between cases with high or low expression of HIF1α protein (Figure 2b and Figure 2d).

Disease-free survival curves of patients treated with postoperative radiotherapy showed that half of the cases with low expression of HIF1α deceased in the first 12 months after surgery, as compared to less than 10% of death in the same period for patients with high HIF1α expression (Figure 2a). Multivariate analysis, considering pTNM, showed that weak expression of HIF1α is an independent prognostic marker, indicating a 3-fold increased risk of death for patients treated with postoperative radiotherapy (HR = 3.41, 1.13–10.34, *p* = 0.029).

Microvessel density was associated with HIF1α expression in operated and irradiated cases (*p* = 0.036), as well as with lower local disease relapse (*p* = 0.001, Table 3).

**Table 3:** Microvessel density relation to HIF1α expression and local disease relapse in operated and irradiated patients.

Microvessel density	HIF1α expression					Local disease relapse				
	Weak		Strong		*p value*	No		Yes		*p value*
	No.	(%)	No.	(%)		No.	(%)	No.	(%)	
≤20	4	(26.7)	0	(0)	0.036	0	0	4	(50)	0.001
20–40	10	(66.7)	10	(66.7)		16	(72.7)	4	(50)	
>40	1	(6.7)	5	(33.3)		6	(27.3)	0	(0)	

After 24 months of follow up, 40 patients were alive, of which 28 (70%) were disease free.

## Discussion and Conclusions

HIF1α protein expression was observed in all squamous cell carcinomas of the lower mouth, being divided into weak and strong expression signals, according to semiquantitative immunohistochemistry staining suggested by Soini et al. [45] and modified by Campos et al. [46].

Our analysis showed a significant relationship between strong HIF1α protein expression and lower local disease relapse (*p* = 0.002) and increased local disease-free survival (*p* = 0.013), suggesting that weak HIF1α expression is an independent risk factor for local disease relapse. Similarly, we have shown a correlation between strong HIF1α protein expression and disease-free survival (Figure 2a, *p* = 0.015) and local disease-free survival for patients that undertook postoperative radiotherapy (Figure 2c, *p* = 0.005). Interestingly, surgery only cases did not show a correlation between HIF1α protein expression and disease-free survival (*p* = 0.700) or local disease-free survival (*p* = 0.391), suggesting an interaction between tumor vascularization and radiotherapy response. Because, no significant relationship between HIF1α expression and tumor size was found, we propose HIF1α expression as a TNM-independent prognostic marker.

Beasley et al. [37] and Fillies et al. [38] have described strong HIF1α protein expression as an independent marker for higher disease-free survival, as well as general survival in patients with head and neck squamous cell carcinomas.

In contrast, lower survival and higher disease relapse has been associated with strong HIF1α protein expression, as reported by Aebersold et al. [39]. However, his work analyzed radiotherapy treated squamous cell carcinomas of the oropharynx, a disease also associated with HPV and therefore with different characteristics [1].

Lin and cols. have described an association between strong HIF1α expression and lower survival in patients with oral squamous cell carcinomas [47]. In this case, immunohistochemistry was quantitative and the scores based on nuclear staining (strong signal attributed to over 60% of immunopositivity). Koukourakis et al. observed a relationship between HIF1α and HIF2α high protein expression and a more aggressive local disease or worse response to carboplatin chemotherapy in squamous cell carcinomas of the tongue, pharynx and larynx [48]. In 2008, a study by Koukourakis et al. showed a relation between HIF1α expression and local disease control in irradiated advanced head and neck tumors, but they did not find the same relation with HIF2α expression [49]. This observation might be explained by the fact HIF1α e HIF-2α can have different functions and tissue specificity, as HIF1α and HIF-2α knockout mice show different phenotypes [50], [51]. Above all, the authors attributed their findings to a higher tumor vascularization.

According to Astekar et al., MVD is directly related with VEGF expression and vascularization of HNSCCs [52]. We have observed that high HIF1α expression is related to higher MVD, probably as a result of VEGF pathway activation, according to previous reports [40]. Moreover, we have detected a correlation between MVD and local disease relapse in patients that underwent post operative radiotherapy. These results suggest that the best disease control is achieved when angiogenesis is stimulated by HIF1α and VEGF expression.

Hypoxia is commonly found in human solid tumors, serving as a selective environment for survival of aggressive cancer cells and as protection from anti-cancer therapies. Commonly, hypoxic tumors are resistant to radio and chemotherapies, since these treatments rely upon the generation of oxygen reactive species to induce lethal DNA damage [53], [54]; however, Zolzer and Streffer [55] showed an increased radiosensitivity of some human tumor cell lines under chronic hypoxia conditions, including squamous cell carcinoma cell lines. This observation was probably due to breakdown of cellular energy metabolism and cessation of cell cycle progression [55].

In comparison, tumors with high expression of HIF1α activate transcription of genes associated with angiogenesis, such as VEGF, therefore it would be reasonable to predict a higher success rate for postoperative radiotherapy in conditions where tissue oxygen concentrations and its reactive species are high, causing a more efficient neoplastic cell death.

It has been shown that increased vascularization of solid tumors can result in higher oxygenation, which together with increased radionuclide uptake show great potential for optimizing treatment strategies, causing better tumor response to therapy [56].

This hypothesis is in complete accord with our results. We propose that head and neck tumors with high HIF1α expression are more sensitive to radiotherapy due to the facilitated generation of reactive oxygen species in a more vascularized microenvironment.

In conclusion, we suggest the utilization of HIF1α protein expression as a squamous cell carcinoma tumor marker to better evaluate the therapeutic options at hand, especially in the decision of postoperative radiotherapy and the establishment of local disease relapse prognosis. For instance, a low expression of HIF1α could indicate the need of more extensive surgical margins. We also suggest that a single immunohistochemistry scoring protocol is adopted, so that results are similarly interpreted worldwide.
